# Dynamic Load Altering Attacks on IoT-Enabled EV Chargers: A Real-Time HIL Co-Simulation Study

**DOI:** 10.3390/s26217000

**Published:** 2026-11-05

**Authors:** Erika De Bardi, Giambattista Gruosso, Giovanna Dondossola, Roberta Terruggia

**Affiliations:** 1 Dipartimento di Elettronica, Informazione e Bioingegneria, Politecnico di Milano, 20133 Milan, Italy; erika.debardi@polimi.it; 2 Transmission and Distribution Technologies Department, Ricerca sul Sistema Energetico (RSE S.p.A.), 20134 Milan, Italy; giovanna.dondossola@rse-web.it (G.D.); roberta.terruggia@rse-web.it (R.T.)

**Keywords:** electric vehicle, dynamic load altering attack, cyber–physical security, hardware-in-the-loop simulation, OCPP, Mininet, OPAL-RT, distribution network, voltage stability, IoT security

## Abstract

Modern Electric Vehicles (EVs) and their charging points (CPs) function as Internet of Things (IoT) nodes embedded in the distribution grid: they continuously sense local electrical conditions, exchange real-time data with the Charging Station Management System (CSMS) via the Open Charge Point Protocol (OCPP), and adjust their power consumption in response to remote commands. This dual role as both a controllable load and a networked sensor makes EV charging infrastructure a high-value target for cyber–physical attacks. This paper investigates the impact of open-loop multi-point Dynamic Load Altering Attacks (D-LAAs) carried out by exploiting this IoT interface on a modified IEEE 13-node distribution feeder hosting twenty CPs at one node. A high-fidelity cyber–physical co-simulation testbed is proposed, coupling a real-time Hardware-in-the-Loop (HIL) power grid model implemented in MATLAB/Simulink and deployed on an OPAL-RT OP4512 platform via RT-LAB, with an emulated ICT network running in Mininet on Ubuntu 22.04. OCPP 1.6 over WebSockets governs CP-CSMS communication, while a TCP/IP interface links the two simulation domains. Two attack scenarios are studied: a coordinated step attack, in which all CP setpoints are first driven to zero and then forced to maximum power (120 kW), and a periodic modulation attack, in which setpoints are rapidly alternated between zero and maximum. Voltages are evaluated at three nodes with varying topological distances from the attack point. In both scenarios, voltage drops reach up to 3.74%. This violates standard voltage-quality margins, demonstrating that corrupting the IoT sensing-and-actuation layer of EV fleets is sufficient to threaten distribution grid stability and underscoring the need for dedicated anomaly-detection and mitigation strategies.

## 1. Introduction

The ongoing digitalisation of distribution networks is progressively blurring the boundary between the power system and the information and communication technology (ICT) layer. Among the actors driving this transformation, Electric Vehicles (EVs) and their associated charging points (CPs) occupy a distinctive position: unlike passive loads, they behave as IoT devices embedded in the grid. Each CP continuously senses its local electrical environment, communicates state and metering data in real time to a Charging Station Management System (CSMS), and dynamically adjusts its power consumption in response to remote setpoint commands. In this sense, a fleet of networked Electric Vehicle Supply Equipment (EVSE) constitutes a distributed layer of grid-edge sensors and actuators, tightly coupled to both the communication infrastructure and the physical power system [1,2].

This dual role—simultaneously a controllable megawatt-scale load and a networked IoT endpoint—makes EV charging infrastructure an attractive and dangerous attack surface. The same communication channel that enables smart charging and demand response can be exploited by an adversary to issue fraudulent commands to multiple CPs simultaneously, effectively weaponising the IoT layer against the grid it is meant to serve. Although this evolution enhances flexibility, optimisation, and demand-response capabilities at the grid edge, it also renders the electrical infrastructure vulnerable to cyber–physical threats [1,2].

The vulnerability of the electrical sector is no longer a theoretical concern. Several real-world incidents have demonstrated the exposure of EV charging infrastructure: in 2024, a major breach exposed 116,000 sensitive records across multiple global EV charging providers, leaking personal details, vehicle data, and authentication tokens. During the Russia–Ukraine conflict, hackers compromised public charging stations to display political messages. Tesla EVs were remotely exploited in 2021, and coordinated attacks against public EVCSs took place in the United Kingdom in 2022. In each case, the attacker’s entry point was the same IoT communication channel through which CPs receive their operating commands. These precedents underscore that EV charging infrastructure represents a critical system characterised by a bidirectional dependency between the cyber and the electrical domains [1].

Within the spectrum of cyber threats targeting charging infrastructure, including Denial-of-Service (DoS) and Man-in-the-Middle (MITM) attacks, Load Altering Attacks (LAAs) are particularly dangerous because they exploit precisely the IoT actuation capability of CPs. Unlike threats that merely block communications or exfiltrate data, LAAs inject false setpoints that corrupt the sensing-and-actuation loop, altering aggregate load demand to disrupt voltage profiles, frequency dynamics, and the generation–demand equilibrium. In the worst case, this can trigger protection actions and cascading failures.

In this study, an EV charging subsystem linked to a modified IEEE 13-node distribution feeder is subjected to open-loop multi-point Dynamic Load Altering Attacks (D-LAAs) to evaluate their impact on voltage. The study leverages a state-of-the-art cyber–physical co-simulation testbed that couples a Mininet-based cyber layer implementing OCPP 1.6 over WebSockets to faithfully reproduce the IoT communication stack of real CPs, with a real-time Hardware-in-the-Loop (HIL) physical model executed on an OPAL-RT platform. Two attack patterns are examined: a coordinated step manipulation of CP setpoints and a periodic modulation of the desired charging power.

The contribution of this work is threefold: it presents a high-fidelity cyber–physical co-simulation methodology that treats EVs as IoT grid-edge nodes; it quantifies the impact of coordinated malicious charging commands on voltage stability; and it demonstrates how such a testbed can serve as a foundation for future monitoring and mitigation strategies targeting the IoT interface of EV fleets. This research extends the foundational co-simulation frameworks and cyberattack analyses presented in [3,4].

The paper is organised as follows. Section 2 illustrates related works. Section 3 details the architectural design of the proposed co-simulation testbed: Section 3.2 describes the physical grid model, Section 3.3 outlines the emulated cyber network topology, Section 3.4 discusses the communication protocols and messaging formats, and Section 3.5 formalises the two attack scenarios. Section 4 presents and analyses the simulation results, and Section 5 draws the concluding remarks and outlines future research directions.

## 2. Related Works and Contributions

In the literature, different works related to testbeds are presented. The primary distinction between these existing solutions and the study herein presented lies in the fact that previous studies typically are not comprehensive of the entire ecosystem, focusing on individual components rather than on the whole.

For instance, Ref. [5] developed an EV testbed specifically designed to perform studies regarding the fluctuations of the frequency caused by renewable energies. While effective for its intended purpose, this testbed lacks the versatility needed for alternative applications such as cybersecurity because it does not model the broader ecosystem.

Similarly, Ref. [6] proposed a framework to simulate only the EV itself. Although this approach shares the real-time features of the present work, it models only the EV’s internal components and fails to capture the holistic system environment.

The work presented in [7] aligns closely with the current study, as it specifically targets the security of OCPP. However, their focus is strictly limited to OCPP vulnerabilities, not taking into consideration other critical infrastructure components. Furthermore, they rely on OpenDSS, which is not a real-time but a steady-state simulator.

These considerations have also been addressed by [8], whose objectives directly parallel those of this study (cybersecurity and its impact on the grid). They reproduced the entire ecosystem comprising the EVCS firmware, realistic EV fleet models, a CSMS, mobile applications, actual communication protocols and a real-time power grid simulator.

Distinct methodological differences exist between their implementation and the approach here proposed:

- First of all, they use Hypersim deployed on an OPAL-RT target, while this work leverages RT-LAB;
- Secondly, for the testbed’s ICT domain, this paper employs Mininet. In contrast, they utilise vSphere, which is an enterprise-grade cloud computing and virtualisation platform. It consolidates physical servers into virtual machines (VMs) to optimise hardware efficiency, reduce operational costs, and centralise IT infrastructure management.

Another article that provides a co-simulation framework is the one developed by [9]. Similar to the present study, the testbed is designed to evaluate the impact of cyberattacks, modelling the electrical grid in MATLAB/Simulink. However, the goal of their study diverges significantly from the one described here. Indeed, they focus on spatially and temporally coordinated cyberattacks focusing on DERs (specifically BESS). Moreover, they model the ICT layer using TrueTime, a specific toolbox designed for MATLAB/Simulink for simulating discrete-event communication networks and real-time control systems. Consequently, they perform a purely mathematical simulation, ideal for evaluating the impact of delays but lacking the emulation capabilities of the presented work. The integration of Mininet allows for recreating a high-fidelity architecture featuring a real TCP/IP stack and actual data traffic.

Similarly, Ref. [10] introduces a co-simulation testbed for cyber–physical power systems. Even if their final goal aligns with this paper (demonstrating the impact of cyberattacks on the electrical grid using the same grid model), they use GridLAB-D for the simulation of the electrical grid. This operates as quasi-steady-state software incapable of capturing fast dynamics. Moreover, for the ICT domain, they use NS-3 (Network Simulator 3), a discrete event simulator wherein attacks must be mathematically modelled in C++, whereas the current work deploys a network emulator. These software tools are orchestrated via HELICS. Although this co-simulation framework does not allow the physical implementation and lacks the fidelity required for structural cybersecurity validation, it excels in large-scale statistical resource planning and quasi-steady-state power flow analysis. By contrast, the proposed OPAL-RT and Mininet testbed achieves a superior cyber–physical fidelity.

Also, Ref. [11] developed a real-time testbed leveraging OPAL-RT. However, they model the cyber layer through internal blocks and abstract control-loop delays, while our work advances the state of the art by implementing a highly realistic, application-level communication infrastructure.

### 2.1. Prior Work by the Authors

The present study builds directly on two previous contributions by the same authors [3,4]. Consequently, the work presented herein significantly advances that previous research by extending the methodology and migrating the framework to another simulation platform.

In particular, with respect to the authors’ previous works, the present study migrates the real-time simulation platform from Typhoon HIL to OPAL-RT/RT-LAB, includes the Mininet network simulator, models a fleet of 20 charging points, and evaluates two open-loop Dynamic Load Altering Attack scenarios.

We use OCPP 1.6 over WebSockets for CSMS–charging-point communication, and a TCP/IP interface connects the cyber and physical layers. This integration enables the study of two open-loop, multi-point attack scenarios using application-layer charging commands and real-time grid simulation.

Furthermore, this architecture is highly scalable and allows for evaluating the impact that cyberattacks can have on the power grid. While this study deploys only one type of cyberattack as a validation case, the framework is flexible, allowing for the assessment of other types of cyber threats.

### 2.2. Contributions of This Work

The analysis above highlights four dimensions along which the existing literature falls short and that define the original contributions of this paper.

1. EVSEs as IoT grid-edge nodes. This work presents an original contribution by explicitly framing CPs as IoT sensing-and-actuation devices and evaluating D-LAAs as attacks that corrupt this IoT interface. Whereas prior work treats the EV charger as a generic load or a protocol endpoint, here the bidirectional CP–CSMS data exchange is modelled at the application level, faithfully reproducing the sensing loop that an adversary would exploit.
2. High-fidelity full-ecosystem testbed. The proposed framework integrates all relevant components—such as OPAL-RT real-time grid simulation, a full charging infrastructure, a CSMS, and a Mininet-emulated ICT network with a real TCP/IP stack—into a single co-simulation environment. No existing work combines OPAL-RT/RT-LAB with Mininet-based OCPP emulation at this level of fidelity.
3. Quantified D-LAA impact on voltage. Two attack scenarios are modelled, executed, and assessed in terms of percentage voltage deviations at three monitored nodes. The results provide concrete, numerically grounded evidence that open-loop multi-point D-LAAs can violate standard grid-stability margins using only the standard IoT communication channel of the CPs.
4. Scalable platform for future mitigation research. The testbed architecture is modular. By separating the physical simulation (OPAL-RT), the network emulation (Mininet), and the application-layer protocol, it provides a reusable foundation for evaluating anomaly detection, authenticated command validation, intrusion detection systems, and other mitigation strategies targeting the IoT interface of EVSE fleets.

## 3. Framework Development and Methodology

The co-simulation testbed, as previously exposed, comprises two distinct but interconnected layers.

- A physical layer that represents the distribution feeder and the EV charging loads in real time;
- A cyber layer that emulates the ICT network, the CSMS, the CPs, and the attacker.

The two domains exchange command and measurement data through a TCP/IP (Transmission Control Protocol/Internet Protocol) co-simulation interface. Figure 1 provides a high-level view of the complete testbed architecture, illustrating how the two layers are structured and interconnected. 

### 3.1. Simulation Ecosystem

The complete ecosystem simulated in this work consists of the following components:

- **CSs**: Each CS comprises two CPs.
- **CSMS**: The Charging Station Management System manages the CSs and communicates with them via OCPP.
- **Protocols**: Standardised communication protocols are used both for the bilateral exchange between the CSs and the CSMS and for interfacing the electrical and cyber domains within the testbed.
- **Power grid**: The power grid is simulated in real time on an OPAL-RT hardware platform.
- **ICT network**: The network architecture is emulated within the Mininet environment.

### 3.2. Real-Time Grid Simulation

The physical layer is executed on a real-time HIL simulator. HIL simulation enables transient and extreme operating conditions to be reproduced safely, while preserving a realistic time base for the interaction with external cyber components.

Nowadays, the market offers different HIL solutions; one example is Typhoon HIL, previously mentioned and used in [3,4,12,13].

In this work, the power grid model is implemented in MATLAB/Simulink (2023b or above) and deployed on an OPAL-RT OP4512 target through the RT-LAB workflow. Developed by OPAL-RT Technologies (Montreal, Quebec, Canada) —an industry pioneer in PC- and FPGA-based real-time simulation since 1997—this platform is purpose-built for testing electrical, electromechanical, and power-electronic systems.

To ensure compatibility with RT-LAB, the model incorporates the required RT-LAB library blocks (OpComm blocks) for input and output data handling. The model architecture is partitioned into two distinct subsystems to comply with RT-LAB compiler requirements: the Master (SM) subsystem and the Console (SC) subsystem. A fixed simulation time step is configured to guarantee deterministic real-time execution.

The electrical network used in this study is a modified IEEE 13-node radial distribution feeder based on [14]. A voltage source supplies the feeder with a nominal line-to-line voltage of 4.16 kV. A transformer between nodes 633 and 634 supplies node 634 at 400 V line-to-line, where 20 CPs are connected. The resulting transformer voltage ratio is 4.16/0.4 kV, rather than the 4.16/0.48 kV ratio specified for the original benchmark. Voltages used to assess the attack impact are monitored at 632, 645 and 675, considering phase 1 as the analysed phase. In Table 1 we report the main parameter values used for the simulation.

The reference feeder is an unbalanced multiphase network comprising three-phase sections and single- and two-phase laterals, with overhead and underground conductors and neutral conductors in the specified line configurations.

Each CP has a maximum specified charging power of 120 kW. With all twenty CPs charging, the initial reference of 60 kW per CP corresponds to an aggregate power of 1.2 MW. The legitimate CSMS reduction to 30 kW per CP gives 0.6 MW. During the attacks, the per-CP commands are changed between zero and 120 kW, corresponding to aggregate commanded charging powers between zero and 2.4 MW. These values refer to the CP fleet and must be distinguished from the total feeder demand, which also depends on the other loads and network losses. The 120 kW rating represents the generic charger for Tesla cars.

The three-phase 500 kV A transformer between nodes 633 and 634 has grounded-wye windings on both sides, a rated voltage of 4.16/0.4 kV, and an equivalent series resistance and reactance of 1.1% and 2%, respectively.

To retrieve simulation values and evaluate the impact of cyberattacks on the grid, sensor blocks were added at specific nodes (highlighted in Figure 2). In particular, nodes 632, 645 and 675 were selected due to their varying topological distances from the attack target node 634. The data generated by these blocks are stored in the MATLAB Workspace via *To Workspace* blocks and subsequently plotted using a dedicated .m script.

To accurately model the real environment, data exchange between the electrical grid simulation and the ICT network emulation was necessary. To achieve this, asynchronous processes had to be implemented inside OPAL-RT using two different blocks. In particular, the OpAsyncSend allows sending values outside the simulation by setting the remote port and IP, while the OpAsyncRecv enables the receiving of data from the external world. To ensure proper functionality, several control blocks were added to bind the unique identifiers of the sending and receiving blocks. Moreover, these blocks allow selecting the desired communication protocol for the data exchange. Specifically, two different protocols can be chosen: TCP/IP or UDP (User Datagram Protocol).

To simulate in OPAL-RT, the time step has to be fixed, and in this study it was decided to set it to $10^{-5}$ s with solver type *ode4 Runge–Kutta*.

### 3.3. Cyber Layer Emulation

The cyber layer has the property of reproducing and emulating the ICT infrastructure. In this study, the network emulation is performed using Mininet, open-source software [15]. Mininet is used to instantiate virtual hosts, links, and an OpenFlow-compliant switch on a Linux-based virtual environment. For this implementation, the software is executed on an Ubuntu 22.04 LTS (Long-Term Support) machine.

The network topology, defined via a custom Python 3.0 script, comprises:

- One CSMS host;
- Twenty individual CP hosts;
- One SDN (Software-Defined Networking) controller responsible for network management;
- One switch acting as the network bridge;
- One attacker host.

Both the CPs and the CSMS are implemented as Python scripts executed inside their respective virtual host terminals.

To reproduce realistic behaviour, CPs electrically connect to the CSMS with staggered delays during the simulation. In the real world, CPs do not connect simultaneously, as their availability depends on EV arrival times. This staggered connection also avoids artificial traffic peaks that could distort the simulation results.

Throughout the simulation, the charging points and the management system exchange data regarding the charging status, the power charging profiles, the charging schedules, and standard OCPP messages such as Heartbeat and BootNotifications. All such messages are exchanged using the standardised OCPP protocol. During the attack scenario, the malicious actor connects to the same communication paths of a target CP and the CSMS, sending false information that disrupts the stability of the ecosystem.

Data exchange between Mininet and OPAL-RT is performed over a TCP/IP interface to ensure deterministic communication with low latency.

### 3.4. Communication Protocols and Network Architecture

As previously mentioned, the communication between the CPs and the CSMS relies on OCPP [16]. This is the globally adopted open standard defined by the Open Charge Alliance (OCA). It ensures seamless interoperability between multi-vendor hardware components and heterogeneous management platforms.

Over the years, different versions of this standard were developed. The initial version was OCPP 1.2 introduced in 2012, followed by version 1.5 in 2013, whereas the most widely used one (OCPP 1.6) was released in 2015. Although improved versions such as 2.0 (published in 2018), 2.0.1 (2020) and 2.1 (2025) have subsequently been deployed, version 1.6 remains the benchmark, and it is the one used in this paper.

OCPP 1.6 is divided into two distinct implementations: OCPP-j, which uses JSON messages transmitted over WebSockets, and OCPP-s, based on SOAP/XML over HTTP(S). The latter, however, suffers from significant per-message overhead. Consequently, in this study, the standard used is OCPP-j, as, compared with SOAP-based OCPP-s, it minimises message overhead and maintains a full-duplex TCP session, offering superior efficiency.

The use of WebSocket messages is not the sole improvement introduced by OCPP 1.6. Key advancements also include smart charging capabilities for load balancing; the introduction of a Local Authorisation List, which enables CSs to memorise locally different user IDs, permitting vehicle authentication and charging in offline mode; and lastly, support for TLS encryption and certificate-based authentication to enhance security. These features are fully integrated into the latest protocol iterations.

### 3.5. Threat Modelling and Load Altering Attack Scenarios

As said before, this work aims to establish a framework for testing cyberattacks and evaluating their impact on the grid. As surveyed in Section 1, the electrical grid is susceptible to different cyberattacks targeting the communication infrastructure. Among them, DoS and MITM represent two fundamental network-layer vulnerabilities [17,18]:

- Denial-of-Service (DoS) attack: A DoS attack is a kind of cyberattack that targets a single system with the goal of attempting to disrupt the normal operations of a network, system, or website by overwhelming it with fake traffic or requests. This resource exhaustion prevents legitimate users from using the services, as the compromised systems become unresponsive while busy handling attack requests. A variant of this attack is the more common Distributed Denial of Service (DDoS), in which the malicious traffic originates from multiple sources simultaneously rather than one as in the DoS.
- Man-in-the-Middle (MITM): An MITM attack is a kind of cyberattack in which a hacker secretly intercepts, relays, and potentially alters communications between two trusting parties. The attacker can execute packet injection or modification attacks, alter critical control setpoints, or forge cryptographic tokens without the awareness of either legitimate entity, thereby subverting the closed-loop control of the physical infrastructure.

The specific threat model considered in this paper is a Dynamic Load Altering Attack (D-LAA) in which the adversary produces voltage instabilities and, potentially, blackouts by synchronising abrupt load variations [1].

In the literature, this type of attack is categorised based on its operational objectives and mechanisms. They can be static or dynamic. While static LAAs seek to physically overload network components (e.g., transmission lines or transformers) by exceeding their thermal capacities or disrupting the generation–demand balance during peak hours, dynamic LAAs (D-LAAs) specifically target system stability. [1,19]. LAAs can be further classified into open-loop and closed-loop, as well as multi- or single-point attacks depending on the number of targeted devices [19].

In this work, two different attack scenarios were performed:

1. In the first attack, when all CPs have halved their charging power, specifically at time 6.6 s, the attacker intercepts the communication path between the CSMS and the CPs and injects malicious commands. Precisely, the attacker initially commands all CPs to stop charging, reducing power to 0 kW. Following a brief pause of 0.8 s, a subsequent command forces all of them to increase their power to approximately their maximum capacity, resulting in a power of 2.4 MW.
2. The second attack is instead a periodic disruption model in which the attacker, upon connecting to the communication channel after all CPs have halved the power (as in the previous attack), issues a cyclic switching command to the CPs, alternating rapidly between 0 kW and 2.4 MW with a frequency of 2.5 Hz. At the end of the oscillatory phase at time 8.8 s, the power is set to the maximum (2.4 MW).

Based on all the previous taxonomies, it is possible to state that the attack can be classified as an open-loop multi-point D-LAA. The attack is open-loop because the adversary manipulates the loads without relying on real-time feedback from the grid state, and multi-point because a plurality of CPs are compromised simultaneously.

Figure 3 summarises the complete simulation execution flow, from the baseline phase through the two attack scenarios up to the measurement and analysis stage.

In this study it was assumed that the adversary had gained privileged access to the CSMS command dispatch layer. This allows the injection of unauthorised charging-setpoint override commands to all twenty CPs connected to the CSMS.

Moreover, as outlined in [16], OCPP supports Security Profiles 1 (Unsecured Transport), 2 (TLS with HTTP Basic Authentication), and 3 (TLS with Client Certificates). In this work, authentication and TLS encryption were omitted to simulate a scenario in which the attacker had already gained access.

## 4. Impact Assessment and Results

To evaluate the impact of the cyberattacks previously discussed, voltage sensors were placed at three nodes of the distribution feeder (632, 645, and 675). To quantify the variations that happen when a threat occurs, a baseline simulation devoid of malicious commands was first simulated as the reference case. The results in terms of total power demand can be seen in Figure 4.

As expected from the simulation setup, the CPs connect with staggered delays, reproducing realistic EV arrival behaviour. During the baseline, the CSMS halves the charging setpoint at a specific moment to mimic a real-world demand-response event and to verify that the CPs correctly track their references. Indeed, the CPs start charging at 60 kW, and then they transition to approximately 30 kW, corresponding to a total demand of 600 kW as can be seen in Figure 4.

The two attack scenarios are then evaluated against this baseline in terms of CP power, node voltages, and voltage variation percentages at node 632.

### 4.1. Voltage Profile Instability

#### 4.1.1. First Attack Scenario

In the first scenario, after the power halving, the attacker forces all twenty CPs simultaneously to 0 kW and then, after a brief pause, to their maximum power of 120 kW, resulting in a total power demand of 2.4 MW. The resulting power behaviour is shown in Figure 5; the voltage deviations relative to the baseline at all monitored nodes are shown in Figure 6.

These results indicate that there are severe complications in the grid. Indeed, the simultaneous load decrease produces a short load-shedding effect and a local voltage rise. Instead, the coordinated load increase produces a much more severe undervoltage transient.

To quantify these variations precisely, percentage increase and decrease were calculated, as shown in Figure 7, based on the simulation values reported in Table 2. While the voltage increase is quite negligible, the undervoltage variations are much bigger. Indeed, in this case, the reduction ranges between 3.46% and 3.74% with the maximum drop observed at node 632.

These values indicate that a coordinated manipulation of charging setpoints can drive the feeder into a degraded operating condition that exceeds normal voltage-quality margins stated by the grid codes. This happens not because of the attack itself that reduces the voltage below crucial limits, but because the undervoltage caused by the attack, added to the normal grid load power consumption, can lead to these crucial conditions. This illustrates how unchecked cyber breaches might endanger the distribution system’s voltage stability.

#### 4.1.2. Second Attack Scenario

In the second scenario, the attacker issues rapid cyclic commands alternating between 0 kW and 120 kW, inducing total power demand oscillations between 0 kW and 2.4 MW as depicted in Figure 8.

Figure 9 presents the comparison in terms of voltage between the baseline and the attack scenario, while Table 2 shows the voltage values measured. As in the previous attack, once the oscillation ceases and the system stabilises at the maximum power (2.4 MW), the grid experiences a severe undervoltage condition.

As quantified in Figure 7, voltage reductions range from 3.46% at node 645 to 3.74% at node 632. These values, combined with normal operating loads, are sufficient to trigger protective relay actions. Overvoltage during the modulation phase is again confined, reinforcing the asymmetry already observed in the first scenario. The problem observed in this last scenario is that there is not only a first negligible overvoltage and then an undervoltage, but for a period of time there is a continuous change between the two conditions, making the scenario more severe.

### 4.2. Systemic Analysis and Cross-Scenario Comparison

Taken together, the results across both scenarios support systemic conclusions.

In both scenarios, the most damaging grid condition is the moment when the aggregate EV load stabilises at the attacker-imposed maximum of 2.4 MW (20 CPs × 120 kW) and voltage margins are violated.

Undervoltage is the dominant risk; overvoltage is self-limiting. Across all nodes and both scenarios, overvoltage from sudden load removal is negligible, while undervoltage from forced maximum loading reached up to 3.74%. This pronounced asymmetry has a direct implication for protection and anomaly-detection design: monitoring systems should be tuned primarily to detect sustained, coordinated load-increase patterns rather than load drops.

Open-loop attacks on the IoT interface are sufficient to violate grid-stability margins. The adversary in both scenarios operates with no real-time knowledge of the grid state. Simply corrupting the OCPP setpoint stream sent to the CP fleet is sufficient to cause voltage drops that exceed standard operating limits. This confirms that the IoT communication layer of EV charging infrastructure is a critical attack surface, and that high-fidelity co-simulation coupling real OCPP protocol emulation (Mininet) with real-time physical dynamics (OPAL-RT) is essential to translate cyber events into meaningful physical risk indicators for grid operators.

## 5. Conclusions and Future Works

This research demonstrates how IoT-enabled EV charging infrastructure might serve as a cyber–physical gateway for distribution grid instability.

The experimental findings reveal that when several CPs work together, even basic open-loop assaults can produce significant voltage degradation. When the attacker synchronises the shift from low demand to high charging power, the most severe impact is seen, triggering large voltage drops. Indeed, in the coordinated step scenario, the voltage at node 632 drops by 3.74%. Conversely, in the periodic modulation scenario, there is a continuous change between the minimal overvoltage and the severe undervoltage. Collectively, these deviations exceed standard security criteria and clearly violate the grid’s acceptable stability margins.

In this paper, we focus mainly on the methodological approach. This can be investigated in more complex grid topologies to analyse the effect of the attacks on different electrical quantities and also investigate different rates of penetration of CPs.

These findings support the high threat level of Dynamic LAAs when they are initiated using IoT-enabled EVSE fleets. Consequently, the developed HIL co-simulation testbed, combining a realistic communication emulation via Mininet with the real-time execution of the power system on OPAL-RT, serves as an indispensable tool. This helps convert cyber events into meaningful grid indicators for developing dedicated monitoring and mitigation strategies at both the network and grid levels.

The same methodology will be used in further research to assess grid-aware mitigation techniques, network-level monitoring and anomaly detection tailored to OCPP traffic, authenticated command validation between the CSMS and the CS, and intrusion detection systems.

Moreover, future implementation will incorporate TLS encryption, also simulating certificate attacks and bypass mechanisms aimed at gaining unauthorised access to the communication channel. This enhancement will ensure a more realistic simulation environment.

## Figures and Tables

**Figure 1:**
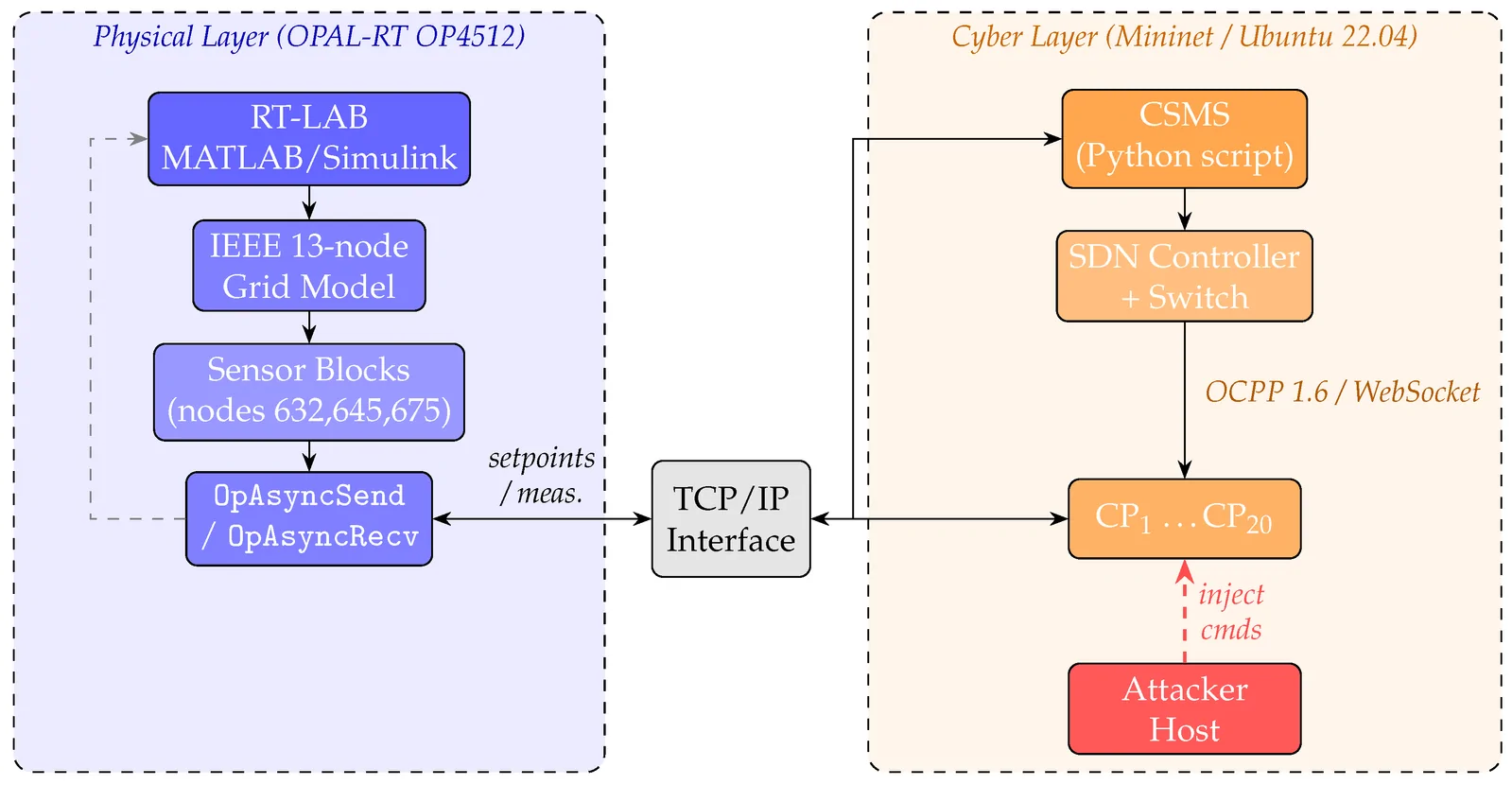
Overview of the proposed cyber–physical co-simulation testbed. The physical layer (**left**) runs the IEEE 13-node grid model in real time on OPAL-RT and communicates with the cyber layer (**right**) through a TCP/IP interface. The cyber layer emulates the ICT network in Mininet, hosting the CSMS, twenty CP nodes, an SDN controller, and the attacker host.

**Figure 2:**
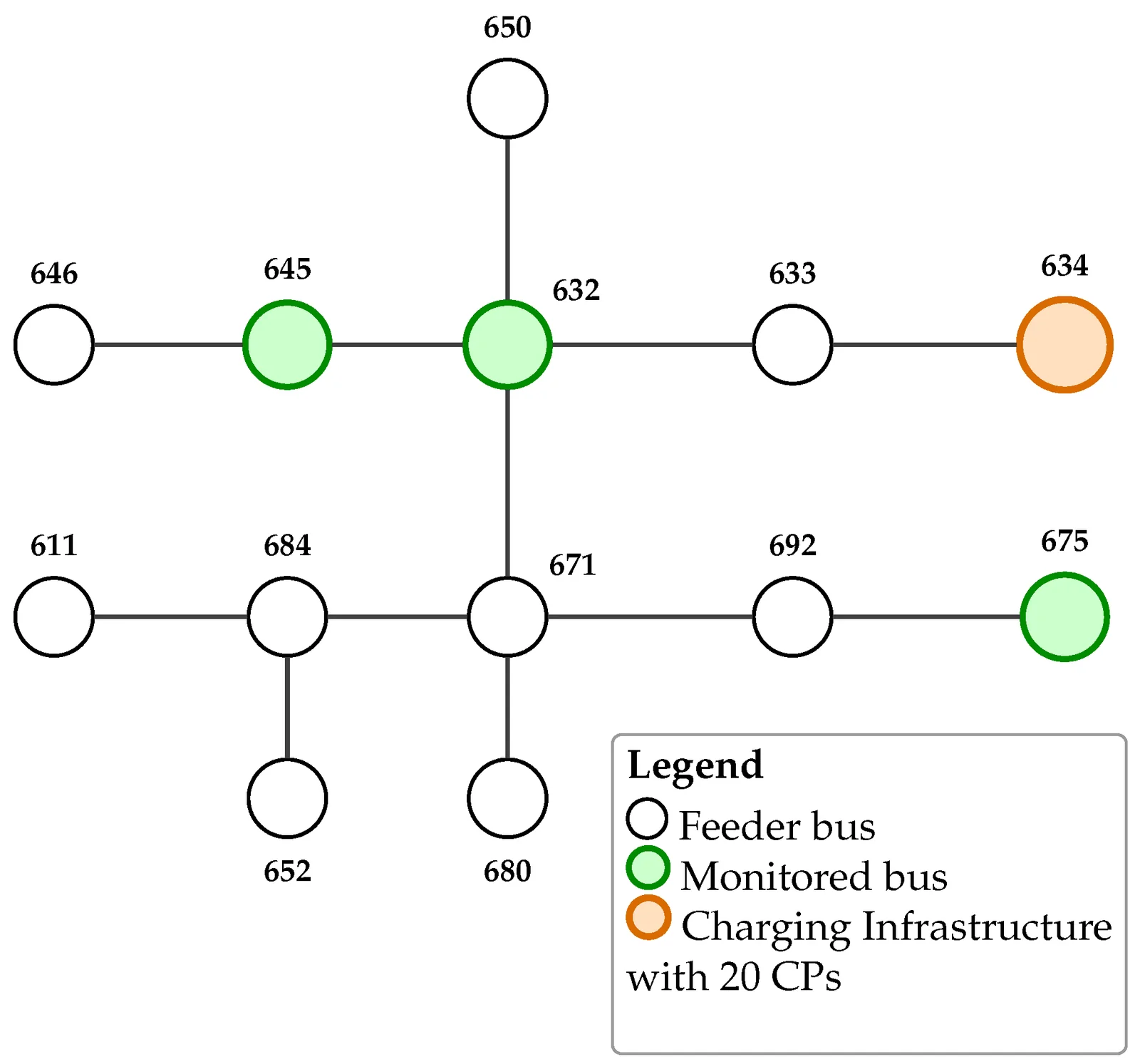
Modified IEEE 13-node test feeder. The orange node (634) hosts the charging infrastructure with the twenty CPs; the green nodes are those monitored during the simulations. It should be noted that, although not depicted in the diagram, a transformer is positioned between nodes 633 and 634.

**Figure 3:**
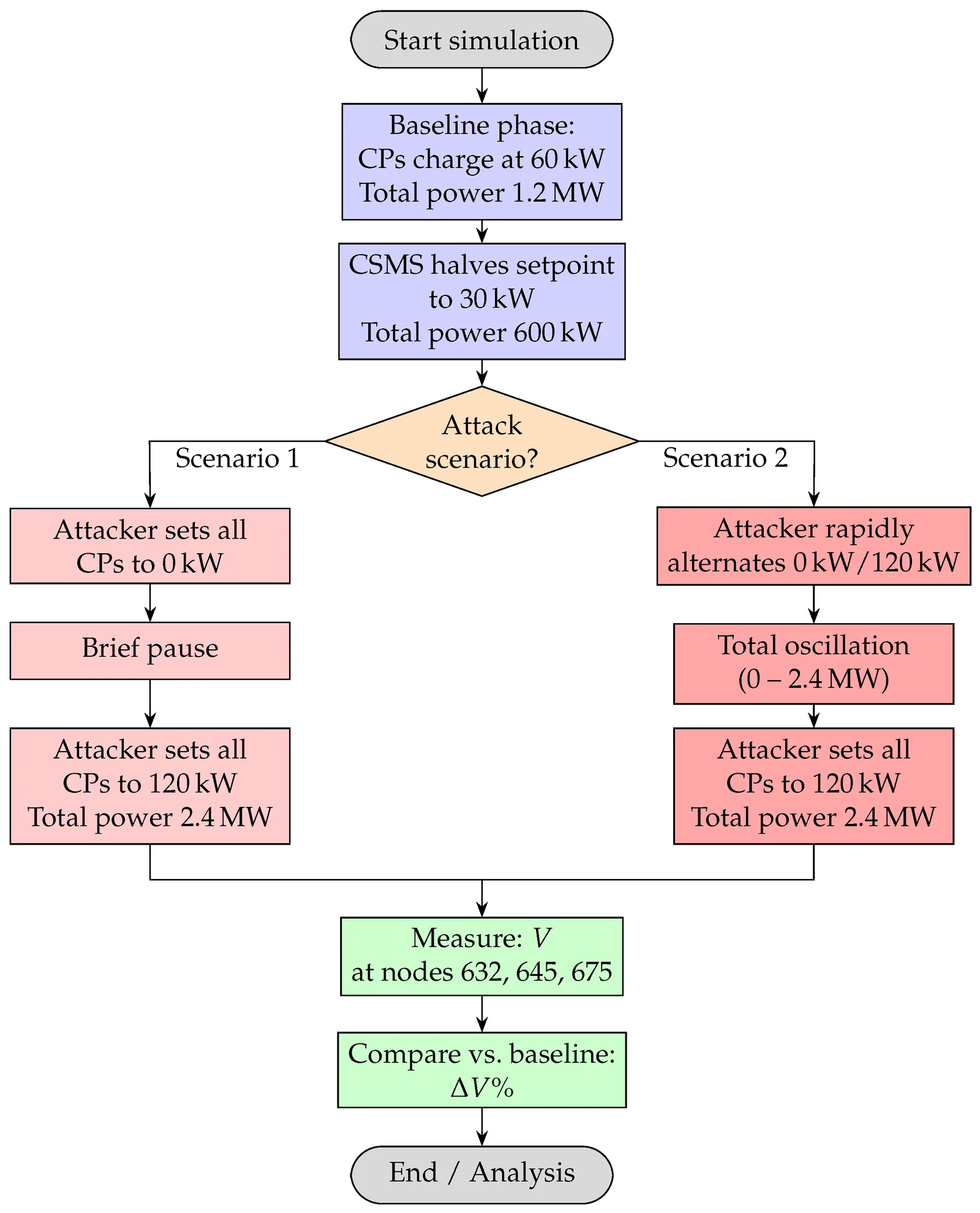
Simulation execution flowchart. After the baseline phase, the two attack scenarios branch: the first scenario performs a two-step setpoint manipulation, while the second one cycles the setpoints before settling at maximum power. Both converge to the same measurement and comparison stage. Although these comparisons are performed independently, they appear in a single branch for visual simplicity. Different colors are for different software modules.

**Figure 4:**
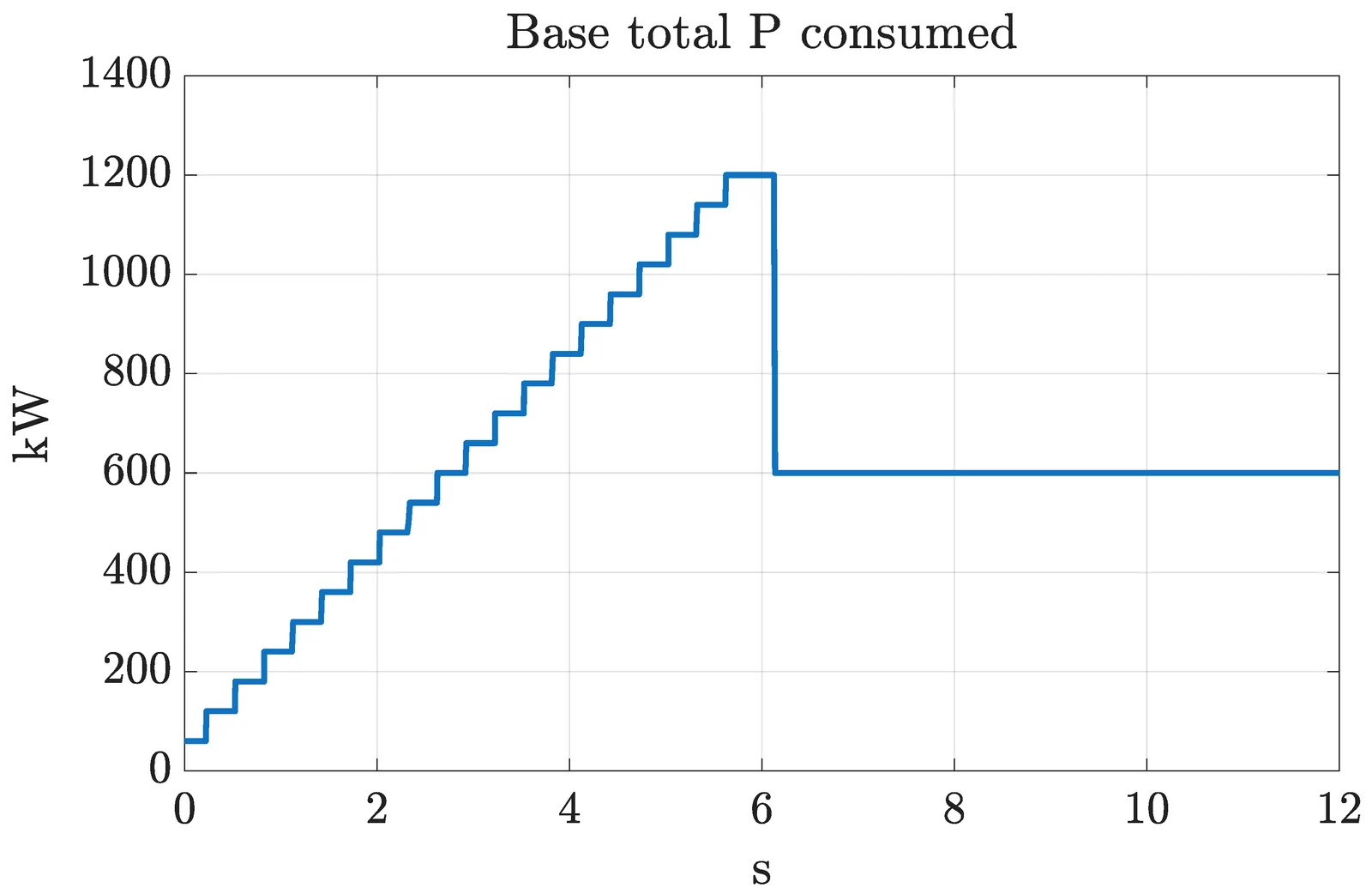
Power profile under normal operating conditions measured at node 634.

**Figure 5:**
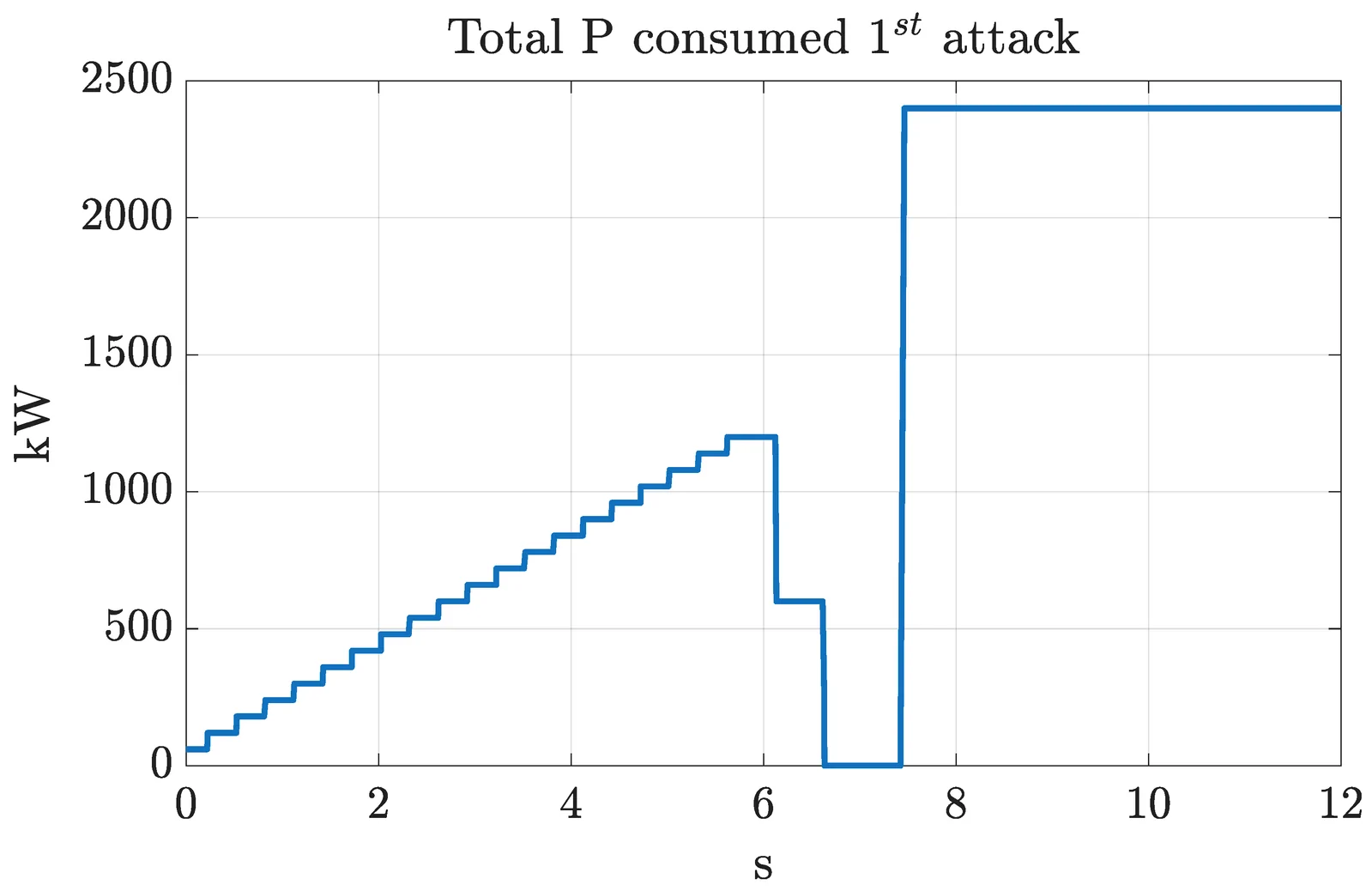
Power profile during the first attack scenario measured at node 634.

**Figure 6:**
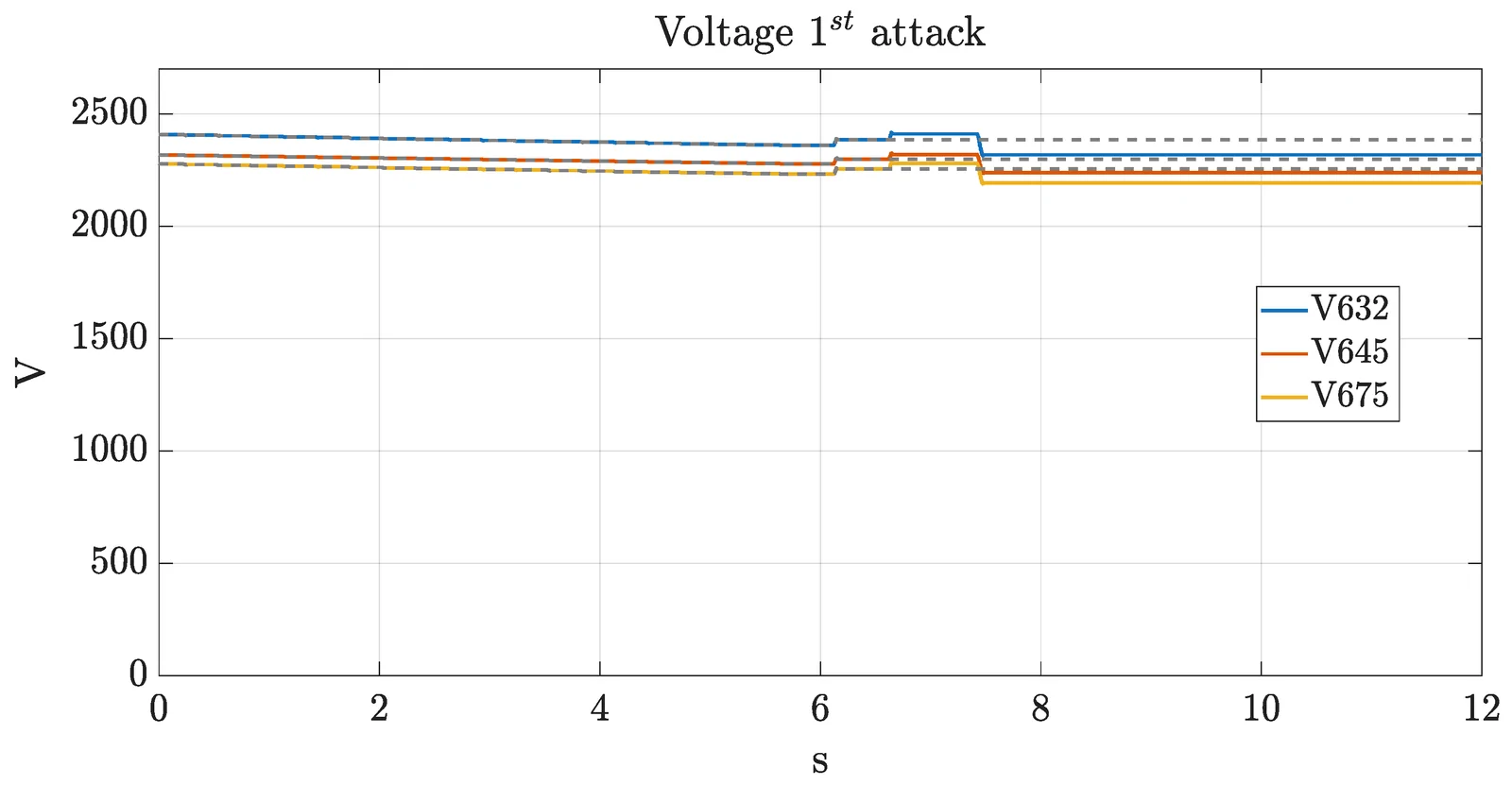
Voltage comparison between the baseline and the first attack scenario at the monitored nodes.

**Figure 7:**
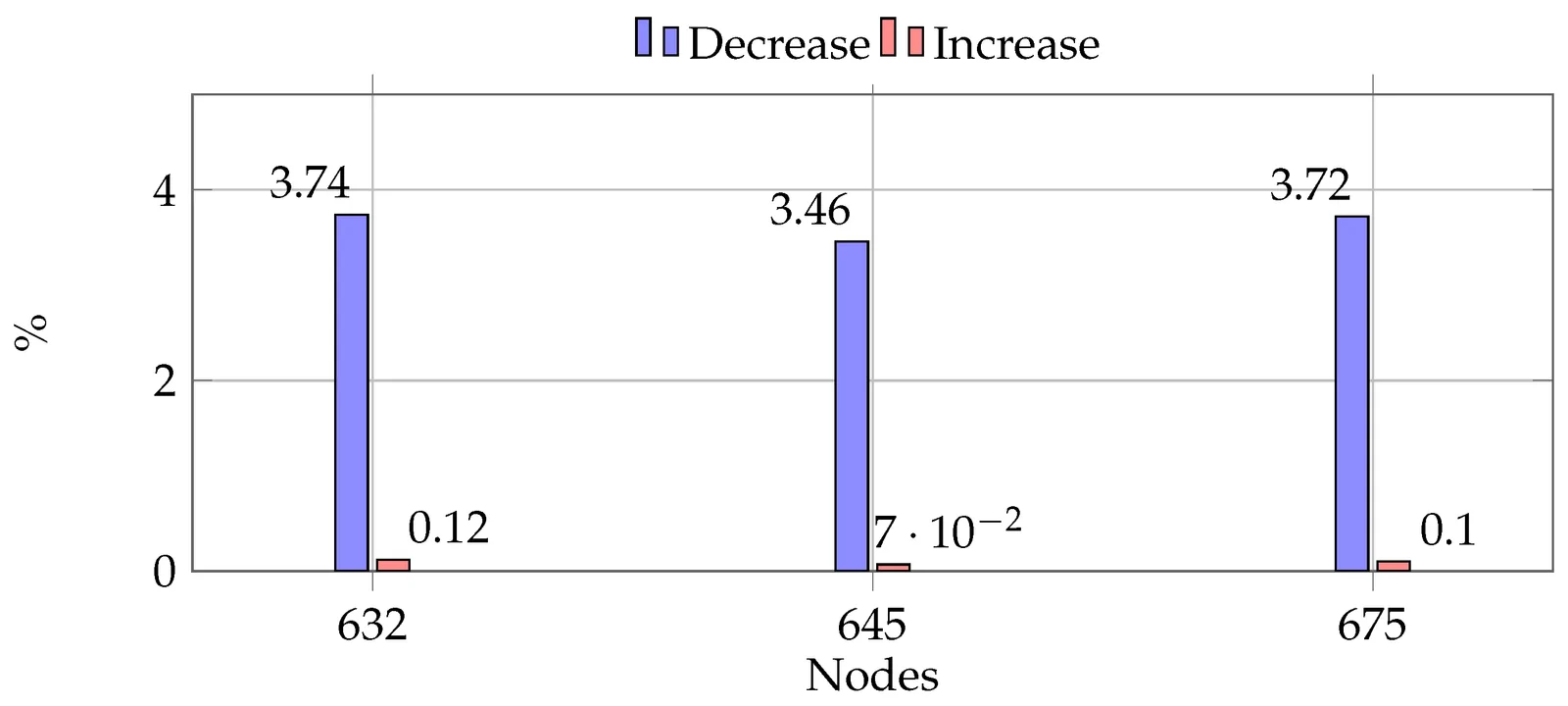
Voltage decreases (blue) and increases (red) measured at monitored nodes during the attack scenarios.

**Figure 8:**
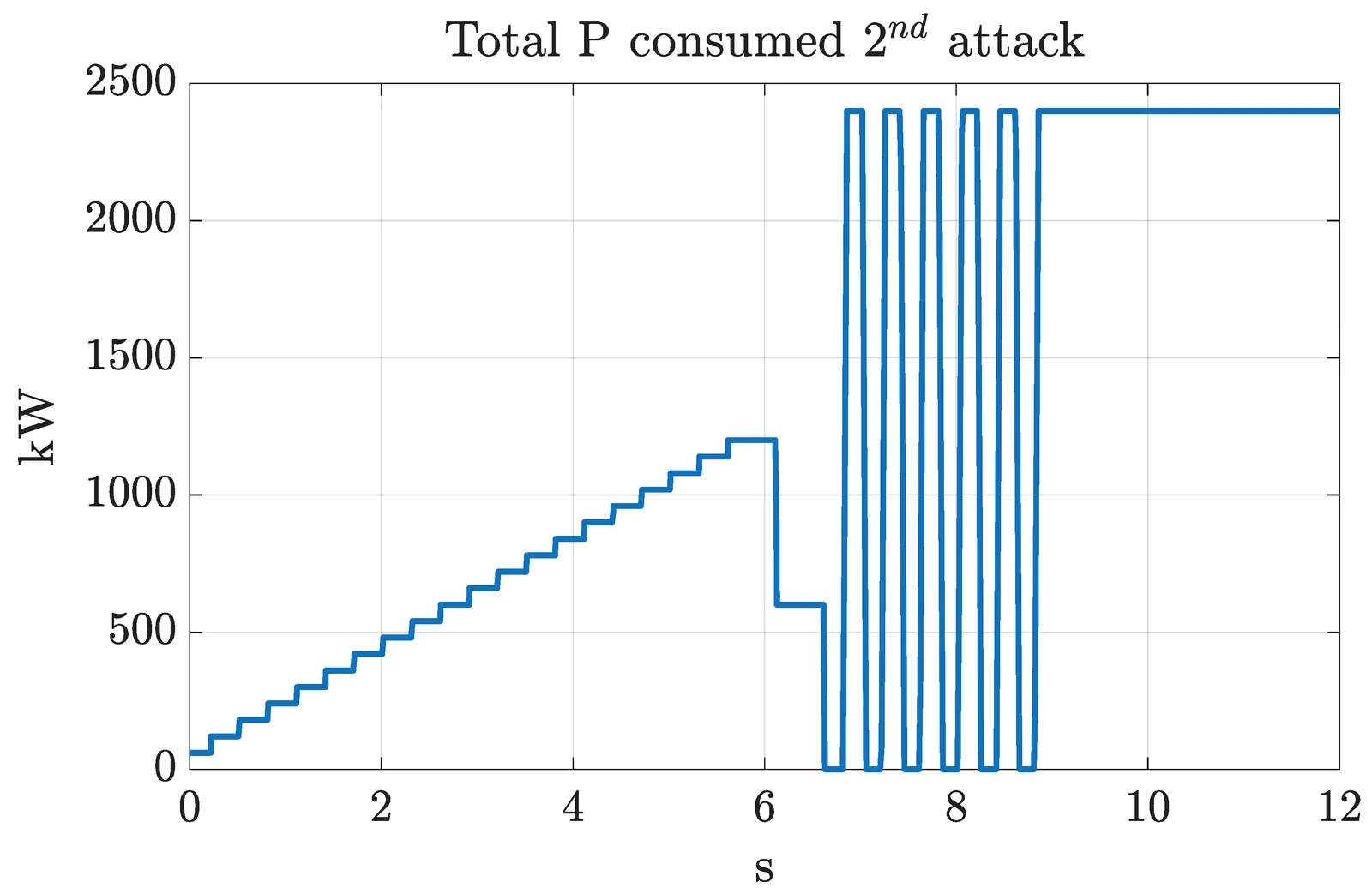
CP power profiles during the second attack scenario measured at node 634.

**Figure 9:**
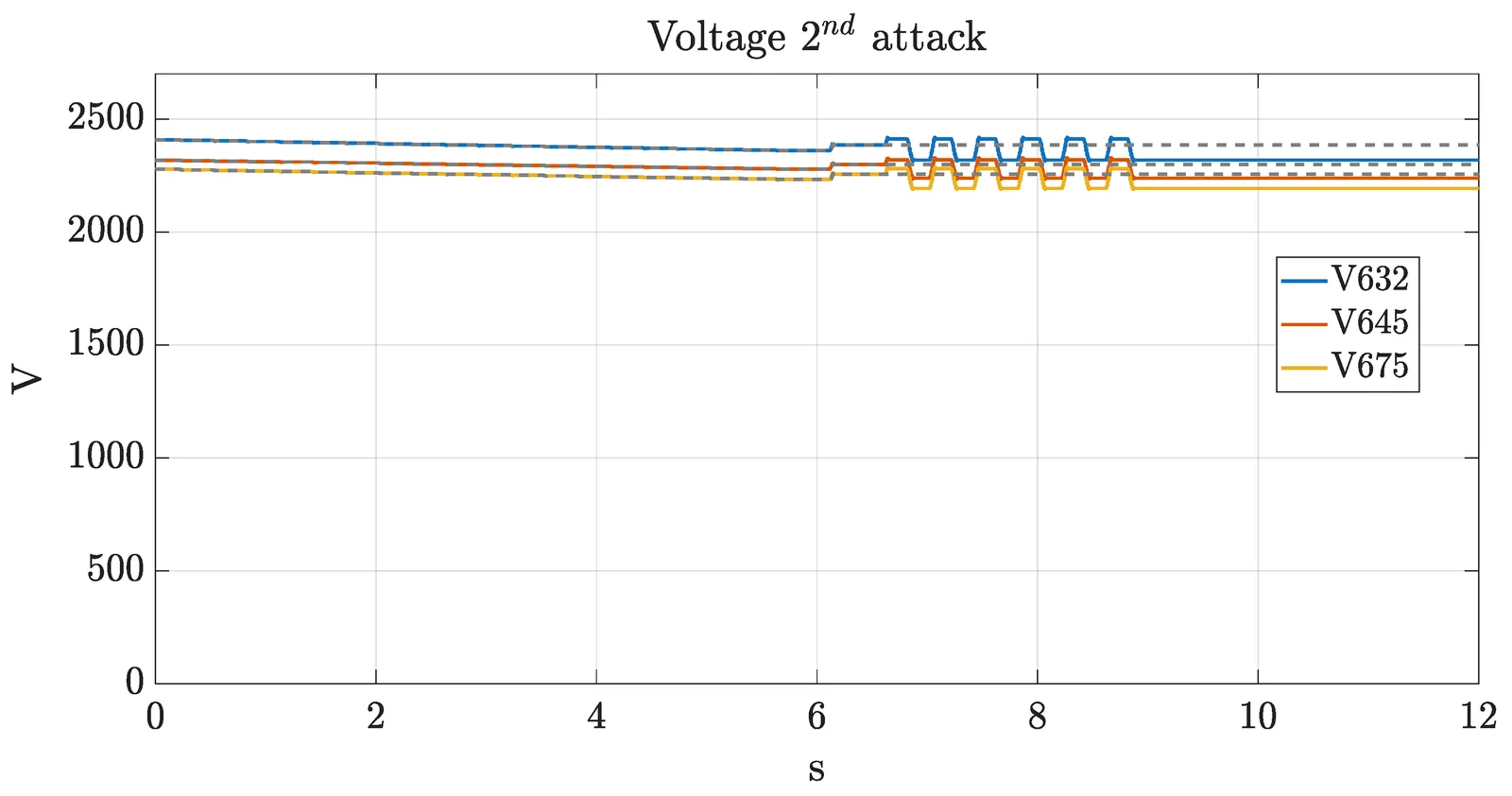
Voltage comparison between the baseline and the second attack scenario at the monitored nodes.

**Table 1:** Electrical and charging parameters specified for the modified IEEE 13-node feeder. The aggregate powers are based on twenty CPs.

Parameter	Value
Feeder supply voltage, line-to-line	4.16 kV
CP connection node	634
Voltage at node 634, line-to-line	0.4 kV
Transformer voltage ratio, nodes 633–634	4.16/0.4 kV
Number of CPs	20
Initial charging reference per CP	60 kW
Reduced charging reference per CP	30 kW
Maximum charging reference per CP	120 kW
Initial aggregate charging reference	1.2 MW
Reduced aggregate charging reference	0.6 MW
Maximum aggregate charging reference	2.4 MW
Monitored voltage nodes	632, 645, 675

**Table 2:** Baseline voltage, undervoltage and overvoltage values retrieved from the simulations.

	Baseline [V]	Overvoltage [V]	Undervoltage [V]
Node 632	2384.83	2411.30	2318.36
Node 645	2299.44	2319.88	2238.09
Node 675	2255.19	2280.03	2192.80

## Data Availability

The data presented in this study are available on request from the corresponding author.
